# Prospective cohort for early detection of liver cancer (Pearl): a study protocol

**DOI:** 10.1136/bmjopen-2024-085541

**Published:** 2024-10-01

**Authors:** Kartikeya Khanna, Eleanor Barnes, Jennifer Benselin, Emma Culver, William Irving, Hamish Innes, Michael Pavlides, DeLIVER Consortium

**Affiliations:** 1 Great Western Hospital Foundation NHS Trust, Swindon, UK; 2 Peter Medawar Building for Pathogen Research, University of Oxford, Oxford, UK; 3 NIHR Oxford Biomedical Research Centre, Oxford, UK; 4 School of Life Sciences, Queen's Medical Centre, Nottingham, UK; 5 Hepatology, Oxford University Hospitals NHS Foundation Trust, Oxford, UK; 6 Translational Gastroenterology & Liver Unit, University of Oxford, Oxford, UK; 7 NIHR Nottingham Biomedical Research Centre, Nottingham, UK; 8 Nottingham University Hospitals NHS Trust, Nottingham, UK; 9 School of Health and Life Sciences, Glasgow Caledonian University, Glasgow, UK; 10 Radcliffe Department of Medicine, University of Oxford, Oxford, UK; 11 Members of the DeLIVER consortium and their associated institutions are listed in the collaborators section, UK

**Keywords:** Hepatobiliary tumours, Hepatobiliary disease, Hepatology

## Abstract

**Introduction:**

Hepatocellular carcinoma (HCC) is the fastest-rising and fourth most common cause of cancer death worldwide. Liver cirrhosis is the largest underlying risk factor for HCC. Therefore, patients with cirrhosis should have regular ultrasound and biochemical screening to pick up early HCC. Early HCC can be cured; more advanced HCCs have limited treatment options and poor prognosis. Current screening methods are suboptimal with poor sensitivity in picking up early disease. In this study, the investigators aim to recruit people with liver cirrhosis into a Prospective cohort for early detection of liver cancer—the Pearl cohort. The investigators believe that by using state-of-the-art tests we can improve the detection of early HCC.

**Methods and analysis:**

This is a UK-based prospective, longitudinal, diagnostic, prognostic, multicentre, non-CTIMP study. Aiming to recruit 3000 patients with liver cirrhosis without a HCC diagnosis, the Pearl cohort will be followed actively for 3 years from recruitment and then passively via registry data for ten years thereafter. Blood and urine samples will be taken and information from routine care will be gathered. These will be used to assess novel diagnostic approaches for the detection early HCC and to develop models to identify those most at risk for developing HCC.

Participants will be linked to national UK health registries to ensure long-term capture of HCC incidence and other relevant endpoints. Approximately 75 patients are predicted to develop de novo HCC within the 3-year follow up period. After this period, the study teams will obtain data on participants for at least 10 years after the last contact. This cohort will help develop an understanding of the incidence of HCC in a UK population stratified by underlying cirrhosis aetiology.

**Ethics and dissemination:**

Ethical approval has been granted by REC and the trial is registered on ClinicalTrials.gov. The results will be published in peer-reviewed journals and presented at relevant meetings.

**Trial registration number:**

NCT05541601.

**STRENGTHS AND LIMITATIONS OF THIS STUDY**

- The only large prospective cohort study to look at patients with cirrhosis across aetiologies while also exploring multiple state-of-the-art and established assays to help facilitate early diagnosis of hepatocellular carcinoma and develop models to help with risk stratification to enhance current screening programmes.
- The Pearl cohort aims to be representative of liver cirrhosis in the UK.
- Multiple academic and industry partners are invested in the study. Their expertise can be leveraged to develop new technologies.
- Data and samples will be collected over time via national UK health registries for long-term outcomes. Variable time of sample collection in relation to development of liver cancer.
- Comprehensive and real-time data capture and integration including the use of daily reporting, statistics, quality control and outlier detection.

## Introduction

Hepatocellular carcinoma (HCC) or primary liver cancer is a major global health problem. It is the fastest-rising and the fourth most common cause of death due to cancer worldwide,^1^ with 854 000 new cases and 810 000 deaths/year.^2^ In the UK, there are ~5500 HCC deaths/year as reported by Cancer Research UK (CRUK). From 1990 to 2015, HCC incidence has increased by 75% globally,^2^,^4^ particularly in high-income regions (including the USA and Europe). It is projected that by 2030, HCC will be the third-leading cause of cancer deaths, surpassing breast, colorectal and prostate cancers.^5^

**Figure 1:**
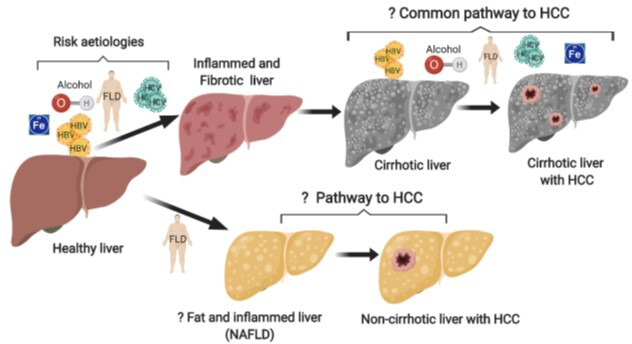
Pathways to hepatocellular carcinoma (HCC)-targeting at-risk populations for surveillance. Alcohol, steatotic or fatty liver disease (FLD), haemochromatosis (Fe) and infection with hepatitis B or hepatitis C virus cause liver inflammation and fibrosis. If left untreated, this can progress to liver cirrhosis and one-third of all patients with cirrhosis will develop HCC. We will target this at-risk population for HCC early detection. Of note however, while 90% of HCC develops on a background of cirrhosis, transformation can occur independently of cirrhosis; in particular, up to 50% of patients with MASLD (NAFLD)-linked HCC will not have cirrhosis. MASLD, metabolic dysfunction-associated steatotic liver disease; NAFLD, non-alcoholic fatty liver disease.

HCC usually arises in patients with advanced liver fibrosis or cirrhosis: 90% of HCC develops on a background of liver cirrhosis^6 7^ (see figure 1); one-third of patients with liver cirrhosis develop HCC in their lifetime.^8^ In the UK, it is estimated that more than 60 000 people have cirrhosis.^9^ Liver cirrhosis can be caused by several different conditions, commonly: viral hepatitis B (HBV) and viral hepatitis C (HCV), alcohol excess, haemochromatosis or metabolic syndrome/obesity/diabetes. Worldwide 75% of HCC is associated with HCV or HBV cirrhosis. The recent advent of effective direct-acting antiviral therapies means that HCV-infected patients with cirrhosis are now cured of infection. However, HCC risk persists, and these patients are retained in surveillance programmes,^10^ with HCC annual incidence 1%–1.8%.^11 12^ The rise in rates of metabolic dysfunction-associated steatotic liver disease (MASLD) (previously known as non-alcoholic fatty liver disease) is increasingly contributing to the underlying HCC annual incidences (0.25%–7.6%).^13 14^ Genetic haemochromatosis contributes to HCC incidence, with UK Biobank data showing high clinical penetrance (>20% with genetic prevalence 1/156).^15^ Since people with liver cirrhosis represent a high-risk group for the development of HCC, these can be targeted for HCC screening for the early diagnosis of HCC (HCC-EDx).

The European Association for the Study of Liver Diseases recommends HCC surveillance in patients with cirrhosis with Child-Pugh A (CP-A), CP-B and CP-C awaiting liver transplant.^16^ Traditionally, HCC screening has relied on liver ultrasound, sometimes combined with alpha-fetoprotein (AFP) measurements. A 6-month interval for screening is standard; shorter intervals have no additional benefit,^17^ while longer intervals are associated with fewer early HCC diagnoses and shorter survival.^18^ Observational and randomised studies of HCC screening in cirrhosis indicate increased detection of HCC at an early stage, increased uptake of curative treatment and survival benefits with surveillance over symptomatic diagnosis, even after accounting for lead-time bias.^19 20^ However, current screening methods have low specificity and sensitivity.^21^,^23^ Indeed, with current surveillance strategies, up to 70% of UK patients have HCC detected at an advanced stage.^24^ Despite this, HCC screening using current existing suboptimal tools has been shown to be cost-effective when HCC incidence is >1.5%/year.^12 25^

Curative HCC therapies (eg, ablation/resection/transplantation) are only offered to patients with early disease (Barcelona Clinic Liver Cancer (BCLC) stages 0 and A). Beyond BCLC-A, the prognosis with all treatment options is poor; chemoembolisation is used for BCLC-B in patients with CP-A, with a mean survival of 2.5 years. Patients with CP-A cirrhosis and unresectable advanced HCC can be offered selective internal radiation therapy with an aim to down-stage the tumours or to bridge systemic medical therapies.^26^ Approved systemic therapies (sorafenib/lenvatinib) improve survival by only 2.3–2.8 months^27 28^ and are used in patients with BCLC-C disease with CP-A. Immune checkpoint inhibitors show antitumour effects in only 15%–20% of patients with HCC.^29^ Therefore, there is an urgent need to develop new strategies to detect HCC at the earliest possible time points when curative strategies may be offered to patients.

Although liver cirrhosis is the major risk factor for HCC, it is unclear why some people with cirrhosis develop HCC, while others do not. HCC is associated with a ‘field effect’ in the cirrhotic liver that predisposes to cancer transformation as evidenced by multiple HCC that arise contemporaneously. However, the pathways to malignant transformation remain unclear. Recent data suggest that malignant transformation in HCC is not primarily driven by archetypal cancer driver mutations.^30^ Furthermore, it is not known if there is a common biological pathway to HCC conversion, regardless of underlying disease aetiology.

Current biomarkers and imaging-driven technologies that aim to identify those most at risk for HCC and to detect HCC at the earliest possible time points are insensitive and outdated. They perform poorly for HCC-EDx and effective strategies for HCC-EDx are, therefore, urgently required. An EDx programme that improved risk stratification and HCC-EDx would significantly enhance the effectiveness of current surveillance programmes.

The Pearl study aims to create a large UK cohort of 3000 patients with liver cirrhosis, at risk for HCC that will be followed prospectively for 3 years, from multiple National Health Service (NHS) UK sites. The study cohort will be used to assess technologies to detect liver cancer at the earliest stages when curative therapies may be applied and to develop new models using a combination of clinical, molecular and genetic data to identify those most at risk for developing HCC. We hypothesise that the combination of state-of-the-art molecular diagnostics and clinical data can be used for the early detection and risk prediction of HCC.

The Pearl cohort will be part of a broader research programme ‘The early Detection of hepatocellular LIVER cancer (DeLIVER)’ which is funded by a 5-year programme grant from CRUK to the University of Oxford. SELiNa (The detection of Small Early Liver cancer with Natural history follow up study) is a sister study to Pearl within the DeLIVER programme, aiming to recruit 250 patients with small HCC. SELiNa is closely linked to Pearl and the data from both cohorts will be used to help evaluate the state-of-the-art assays being tested (see the ‘Objectives and outcome measures’ section).

## Methods and analysis

### Study design

Pearl will be a UK-based prospective, longitudinal, diagnostic and prognostic, multicentre study. This study is observational in nature and does not require any interventions (ie non-CTIMP (clinical trial of investigational medical product) or changes to standard clinical care.

The investigators aim to enrol 3000 participants from specialist liver clinics from multiple NHS hospital trusts in the UK. These trusts will be those that are part of the National Institute for Health Research Clinical Research Network (NIHR CRN) and NHS Research Scotland. Participants will have a diagnosis of liver cirrhosis and no history of HCC. All will be attending specialist services for management and treatment of their disease and routine screening for HCC. Enrolled participants will be asked to complete at least three visits; a baseline visit plus two further follow-up visits over a period of 3 years. An unscheduled visit may occur if a participant is diagnosed with HCC. During each study visit, participants will have samples taken and clinical data and information collected. Wherever possible, these study visits will be timed to coincide with routine clinic visits to minimise the burden for participants in terms of travel to the hospital, time spent in clinics, additional blood tests and other assessments.

**Figure 2:**
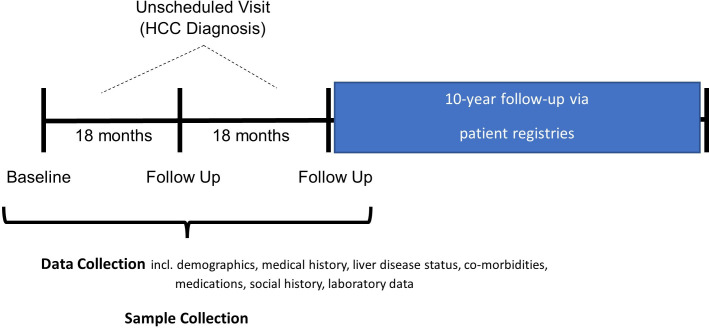
Pearl visit schedule highlighting prospective follow-up of participants. HCC, hepatocellular carcinoma.

Long-term follow-up of medical records from national health registries will be conducted for at least 10 years after the end of the active follow-up phase. Data obtained from NHS England and other patient registries will include decompensation episodes, HCC diagnosis, liver transplantation and death (see figure 2).

### Recruitment

Participants for the Pearl study will be recruited at specialist clinics at NHS Hospital Trusts. Participants will be identified and screened to the inclusion and exclusion criteria by a member of the clinical care team or research team to ensure eligibility for the study. They will then be approached and given both written and verbal information regarding the study. Written informed consent will be obtained from every participant prior to enrolment at a clinic appointment. Eligible patients with cirrhosis may only attend clinic appointments every 6 months; therefore, it is anticipated that participants will be approached face-to-face about the study, consented and have their baseline visit on the same day. Patients who are eligible for the study but decline to participate will be logged into the site-specific study logs. This will allow for some level of review and analysis when looking at the final data set.

22 Pearl recruiting sites also participated in the MRC-funded STOP-HCV Cirrhosis Study and it is highly likely that surviving Cirrhosis Study participants will still be attending clinic and will be eligible to enrol into the Pearl study. STOP-HCV has a large dataset of deep-phenotyped participants with HCV-related cirrhosis successfully treated for HCV, followed prospectively since 2015 with annual blood sampling; we will ask permission of STOP-HCV Cirrhosis Study participants who are eligible and are recruited into Pearl to store their STOP-HCV study number for the purposes of data linkage.

### Participants

The investigators will recruit participants with Child- Pugh A or B cirrhosis with no current or historical diagnosis of HCC. Participants can be recruited to other interventional studies for treatment of cirrhosis or prevention of HCC prior to or during participation in the Pearl study.

### Inclusion criteria

1. Patients of all genders, age >18 years.
2. Participants are willing and able to give informed consent for participation in the study.
3. Evidence of cirrhosis Child-Pugh A or B (see table 1), with an underlying aetiology of at least one of the following:
  1. Chronic HBV infection.
  2. Chronic HCV infection.
  3. Alcoholic liver disease.
  4. MASLD.
  5. Haemochromatosis.

**Table 1:** Cirrhosis diagnosis definition

Criteria A or one of criteria B		
Criteria A	Criteria B	
Histological Assessment(Ishak stage 5 or 6)	Validated non-invasive markers of fibrosis including FibroScan, APRI score >2 or ELF score >10.48 or Fibrotest score >0.73.	FibroScan readings should be assessed by aetiology: HBV: ≥10 kPa HCV: ≥14.5 kPa ALD: ≥19.5 kPa MASLD (NAFLD): ≥15 kPa Haemochromatosis: ≥12 kPa
	Evidence of varices at endoscopy or imaging in the context of a patent portal vein	
	Definitive radiological evidence of cirrhosis (ie, nodularity of liver and splenomegaly on ultrasound/CT)	

APRI score: AST to Platelet Ratio Index score

ELF score: Enhanced Liver Fibrosis score

ALDalcoholic liver diseaseHBVhepatitis B virusHCVhepatitis C virusMASLDmetabolic dysfunction-associated steatotic liver diseaseNAFLDnon-alcoholic fatty liver disease

### Exclusion criteria

1. Diagnosis of current or historical HCC.
2. Liver transplant recipients or patients on active listing for liver transplantation.
3. Child-Pugh C cirrhosis.
4. In the view of the clinician, if the patient has a comorbidity likely to lead to death within the following 12 months.
5. In the view of the clinician, if the patient is not thought to be suitable for HCC surveillance.

### Protocol procedures

Each participant will have three scheduled study visits: one baseline visit and two subsequent follow-up visits over a 3-year period. During the baseline visit, participants will be consented, enrolled into the study and allocated a unique study number. Their demographics, anthropological measurements (height, weight and body mass index), medical history and previous clinical imaging data will be collected. They will also have blood and urine samples collected and stored. All data and biological samples will be recorded against their unique study number.

During the two subsequent scheduled follow-up visits, 18 months after previous visits (which will be timed to coincide with routine clinic appointments where possible) there will be further collection of data as above (including blood and urine samples). If participants are unable to attend a face-to-face appointment the participant may be contacted via phone or email to respond verbally or online to a questionnaire (for medical history and demographic data).

Participants may have unscheduled follow-up visits. This will occur if the participant is diagnosed with HCC and should happen as close as possible to the date of HCC diagnosis. Participants will have the same data points, including blood and urine samples collected on this visit. Blood and urine samples will need to be collected prior to initiation of any treatment for HCC. Follow-up will then continue as per the standard protocol to ensure outcome data and samples are collected (see figure 2).

If the participant received a liver transplant, they would remain part of the study but no further follow-up visits or samples are required. If the participant dies the cause of death will be recorded clearly.

Beyond the 3-year active follow-up process, NHS England data from participants will be obtained for at least 10 years after the last participant contact. This passive follow-up will involve no direct participant contact.

### Sample handling and analysis

Participants will have blood and urine samples taken on each study visit (both scheduled and unscheduled). The research samples will be collected, have initial processing in the local laboratory and stored (as per sample collection protocol) prior to transfer to Oxford University labs.

Samples are handled and stored as per Human Tissue Act requirements; all samples are labelled with a unique visit ID and will not contain any personally identifiable information. All future subdivisions of the sample will be allocated a new unique barcode. Linking of clinical data to the samples will be possible using unique participant study numbers and visit ID numbers.

The blood samples will be tested for state-of-the-art biomarkers including (but not limited to):

- Detection of epigenetic and genetic mutations and copy number variations in circulating tumour DNA.
- Polygenic risk scores derived from germline genetic sequencing.
- Detection of autoantibodies to tumour-associated antigens.
- Protein biomarkers include the L3 isoform of AFP and des-gamma-carboxyprothrombin.
- Viral sequencing (HBV and HCV).
- Proteomics and metabolomics.

The urine samples will be analysed for:

- Proteomic and metabolomic profiling including steroid metabolic signatures in urine.

Analyses will be carried out in laboratories within the University of Oxford or with collaborators at other academic institutions. Participant consent for these analyses and storage and future use of remaining blood and urine samples for yet unspecified ethically approved research purposes will be sought. Samples will be retained indefinitely.

### Objectives and outcome measures

The primary objective of Pearl is to determine the performance characteristics of novel state-of-the-art diagnostic approaches for the early diagnosis of HCC in study participants who are diagnosed with HCC by conventional means. For each novel diagnostic approach (whether as a single test or combination of tests) the outcome measures will be the sensitivity, specificity, and positive and negative predictive values in terms of HCC diagnosis for each test. The diagnostic approaches to be tested are as outlined in the section ‘Sample handling and analysis’. Evaluation of the outcome measures will begin once 50 cases of HCC have accumulated within the Pearl cohort.

**Figure 3:**
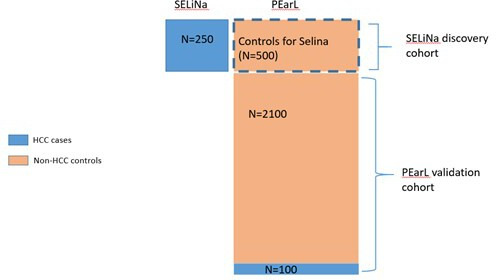
Schematic outlining how the Pearl cohort will be used in conjunction with the SELiNa cohort. HCC, hepatocellular carcinoma.

The primary objective outlined above will be evaluated in conjunction with data from the DeLIVER sister study SELiNa. The Pearl cohort will be divided and used as part of the SeLiNa discovery cohort (providing non-HCC cirrhosis controls), and as the validation cohort (see figure 3).

A key secondary objective of Pearl is to develop prognostic models for ‘risk-stratifying’ patients with cirrhosis according to their future risk of HCC. This could enable clinicians to personalise HCC surveillance. The models developed will be evaluated primarily in terms of their discriminative ability (ie, ability to differentiate individuals who develop HCC from those who do not), measured via the Concordance Index (C-Index). In this context, the C-index will indicate the degree to which individuals who develop HCC have a higher risk score than those who do not.

An additional secondary objective includes quantifying the cumulative incidence of HCC among people with cirrhosis and determining how this varies by underlying cirrhosis aetiology in a UK setting. The cumulative incidence of HCC (stratified by HCC aetiology) will be measured at 1, 3 and 5 years postbaseline.

A further secondary objective will be using the Pearl cohort to identify patients at high risk of developing HCC using the AMAP clinical risk score, for the development of novel MR imaging as part of the separate AMULET study.^31^

### Statistics and analysis

#### Primary objective (evaluate diagnostic performance of early diagnosis assays)

The investigators will assess the performance of selected detection assays for diagnosis of early HCC. This will be achieved by comparing assay values for early HCC cases with those of non-HCC controls (using the SELiNa cohort data). The performance characteristics of primary interest for each assay will be:

1. Sensitivity (ie, proportion of HCC cases with a ‘positive’ result).
2. Specificity (ie, proportion of non-HCC cirrhosis cases with a ‘negative’ result).
3. Positive predictive value (ie, probability of having HCC for individuals with a ‘positive’ result).
4. Negative predictive value (ie, probability of not having HCC for individuals with a ‘negative’ result).

The area under the receive operating curve, derived from an assay’s sensitivity and specificity, will also be calculated as a summary measure.

As well as quantifying the performance characteristics of each assay in isolation, we will also assess the degree to which performance characteristics may be augmented by combining information from multiple assays into a single ‘multivariable’ model. Simplest methods will be considered first, considering increasingly complex methods if they provide value, for example, logistic regression, random forests, support vector machines.

#### Secondary objective (prognostic models)

A key secondary objective is to develop prognostic models (ie, risk scores or risk calculators) that estimate a cirrhosis patient’s individualised risk of developing HCC in the future. The investigators will approach this in two main ways. First, developing static risk models that incorporate biomarker data collected at a single time point only (ie, study enrolment). Second, building dynamic models that leverage the serial data collected for Pearl participants to estimate individualised HCC risk. As with our primary outcome, our focus will be on developing models that combine multiple prognostic factors/detection assays.

In general, we will endeavour to align all statistical analyses with ‘best practice’ guidelines established in the TRIPOD (Transparent Reporting of a multivariable prediction model for Individual Prognosis or Diagnosis) statement.^32^

### Sample size

**Figure 4:**
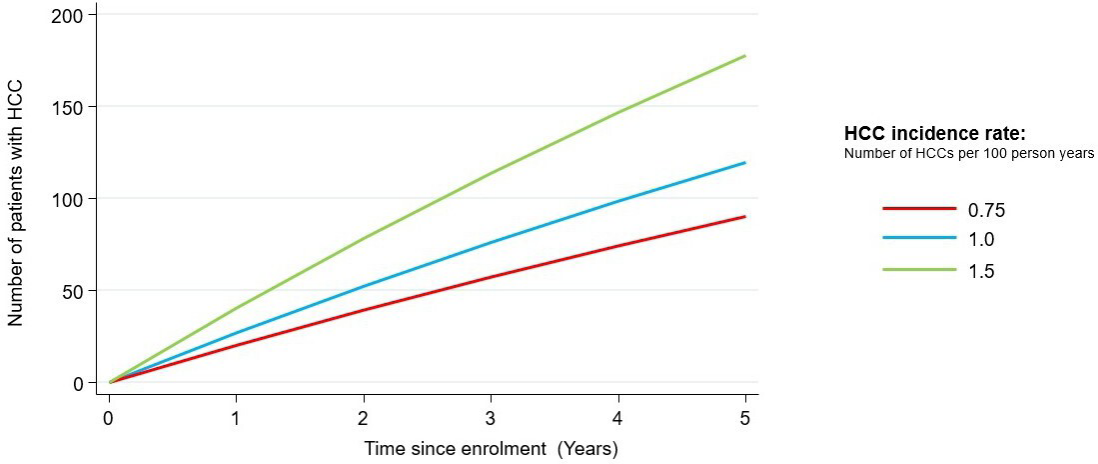
Number of patients expected to develop HCC in the Pearl cohort, according to time since enrolment and HCC incidence rate (N.B. These estimates assume an overall sample size of 3000 patients with 10% attrition. All-cause mortality is estimated at 5% per year). HCC, hepatocellular carcinoma.

The study will aim to recruit 3000 patients with cirrhosis over 30 months of specified aetiologies. There is an expected ~10% drop-out rate, leaving a final cohort size of 2700. There are currently more than 60 000 people living with cirrhosis^9^ and many of the recruiting centres have several hundred patients under annual surveillance. The study has received 23 letters of support from these centres which demonstrate the national interest in this study and demonstrate that the recruitment target is realistic. It is anticipated that this cohort will yield ~90–180 incident HCC cases over, for example, a 5-year time frame, depending on the HCC incidence rate observed (figure 4). Based on the rule-of-thumb of 10 events per variable, this would provide scope to include ~10–20 prediction parameters in our model, for this time horizon. Many of the parameters to be evaluated as part of the model will be selected depending on data generated from exploratory laboratory assays. The study has been adopted onto the NIHR CRN portfolio facilitating national recruitment.

### Data handling

All study data will be entered into an Electronic Data Capture (EDC) system called REDCap or onto a paper source document for later entry into EDC if direct entry is not available. REDCap is a well-supported EDC that is widely used within the University of Oxford and further afield. It has been previously implemented in large national and international trials. The REDCap database is a secure database, fully compliant with Good Clinical Practice (GCP), European Union and UK regulations, allows a full audit trail for tracking data manipulation and user activity and is backed up on a frequent basis. The database resides on secure firewalled servers maintained by the University of Oxford and located within the University of Oxford Medical Sciences Division secure network. Only members of the study teams have access to REDCap via individual username and password which only allows access to their specific data. Additional information may be recorded on a separate paper source document or electronic files.

The participants will be identified by a unique study-specific number in any database and other electronic or paper documents apart from the code break document (site enrolment log) and consent forms which will be stored securely in confidential conditions. With participant permission, we will store names, telephone numbers and email addresses for the purpose of facilitating telephone and/or email follow-up visits. Paper documents containing personal information will be stored in locked cupboards at study sites and access will only be given to the authorised persons. Electronic documents containing personal information will be stored on secure servers with access restricted to the local study team in the case of the enrolment log or the Pearl Management Group which can access participant contact details.

Personal information such as contact details will be destroyed no later than 12 months after the end of the study unless the participant has agreed to be contacted for future research in which case the personal information will be transferred to another secure database. The personal identifiers (name of participant) contained in consent forms will be stored or accessed for up to 5 years after study end by the study sites, after which time the custodian will agree on a date for destruction and it will be destroyed confidentially. Fully anonymised research data obtained from assays and analysis will be stored and accessed indefinitely for the purpose of continuous publication opportunities. To facilitate long-term data collection of mortality and cancer-related endpoints, study investigators may request patient information from NHS England and/or other patient registries. Of note, the NHS and CHI numbers of participants will be recorded in REDCap for the purpose of linkage with patient registries. Data returned to the research team will only contain the unique study-specific number.

## Ethics and dissemination

Pearl will be conducted in accordance with the principles of the Declaration of Helsinki, relevant regulations and GCP guidelines.

Approval for the study has been obtained from HRA and REC. The study has been registered on ClinicalTrials.gov (NCT05541601). As a non-CTIMP, observational study, there will be minimal risks associated with participation. The risks involved would be those of venepuncture.

This protocol is version 3.0 (30 June 2023): there have been two substantial amendments to the protocol which have both received HRA and HCRW approval.

The results of the study will be published in a peer-reviewed journal and presented at relevant conferences.

### Patient and public involvement

Input into the study design as well as the generation of protocol and participant information sheet has been sought from patient groups (as part of the British Liver Trust and the Hepatitis C Trust) and patient representatives are part of the DeLIVER programme Management and Steering Committee which will discuss the management of all studies under the umbrella of the DeLIVER consortium, including the Pearl study.
