# Supplementary material for: Prospective cohort for early detection of liver cancer (Pearl): a study protocol
Source: BMJ Open. 2024 Oct 1;14(10):e085541. doi: 10.1136/bmjopen-2024-085541 (PMC11448217; doi:10.1136/bmjopen-2024-085541)
Supplement: Supplementary file 1 [file bmjopen-2024-085541.reviewer_comments.pdf]

PEER REVIEW HISTORY

BMJ Open publishes all reviews undertaken for accepted manuscripts. Reviewers are asked to complete a checklist review form and are provided with free text boxes to elaborate on their assessment. These free text comments are reproduced below.

ARTICLE DETAILS

**TITLE (PROVISIONAL)**	Prospective Cohort for Early Detection of Liver Cancer (Pearl): A Study Protocol
**AUTHORS**	Khanna, Kartikeya; Barnes, Eleanor; Benselin, Jennifer; Culver, Emma; Irving, William; Innes, Hamish; Pavlides, Michael; Consortium, DeLIVER

VERSION 1 - REVIEW

**REVIEWER NAME**	Karanicolas, Paul
**REVIEWER AFFILIATION**	Sunnybrook Health Sciences Centre, Surgery
**REVIEWER CONFLICT OF INTEREST**	None
**DATE REVIEW RETURNED**	09-Mar-2024

**GENERAL COMMENTS**	This manuscript presents a protocol for a prospective cohort study of 3000 patients with liver cirrhosis, aiming to assess novel diagnostic approaches for the detection early HCC. Participants will be linked to national registries and followed for three years. 75 patients are predicted to develop de- novo HCC during the study period. Long-term follow-up will continue for 10 years.<br><br>Overall, the study is well-designed and the manuscript provides sufficient details of the protocol. I have the following minor comments that the authors might consider:<br><br>What is the rationale for excluding patients who do not have one of the five specified aetiologies of HCC? The diagnoses listed are the most common but patients with other causes of cirrhosis are still at increased risk of developing HCC and could contribute rich information to this study.<br><br>I'm concerned exclusion criteria #4 "In the view of the clinician, if the patient has a co-morbidity likely to lead to death within the following 12 months" is too subjective, and could limit enrolment or generalizability of the ultimate cohort. I understand the rationale, to limit enrolment of participants who will die during the study period and thus not contribute meaningful information to the analysis, but I wonder if the downsides of this criteria outweigh the potential benefit. The same applies to criteria #5: "In the view of the clinician, if the patient is not thought to be suitable for HCC surveillance." At a minimum, participants who are excluded on this basis should be recorded and reported in the ultimate manuscript so that clinicians can interpret the applicability of the findings.<br><br>The diagnostic criteria for cirrhosis (table 1) seem like they would include a patient with left-sided portal hypertension (for example, a patient with chronic pancreatitis causing splenic vein occlusion and resulting varices). I know this is not the intent, but the authors might

	consider revising this to avoid inadvertent inclusion of participants without cirrhosis.<br><br>The sample size calculation estimates 75 participants developing HCC over the timeframe of study, then goes on to say that allowing 10 events per variable, up to 10 prediction parameters will be included. Shouldn't this read 7 variables to be included, rather than 10? Ideally the authors would state which variables they plan to include in the model, or some description of how they will select the variables. The analysis section in general is very short and lacks many details, this really should be expanded to provide more information on the analytic plan.<br><br>The study is non-interventional, but this might provide a nice opportunity to identify patients upon diagnosis of HCC who might be candidates for interventional trials. In particular, if patients are found to have more advanced HCC during surveillance (clearly not the intent), there will likely be other interventional trials that they qualify for. The authors might consider working with existing collaborative groups to provide trial opportunities for patients newly diagnosed with HCC as part of the cohort study.

**REVIEWER NAME**	Cross, Tim
**REVIEWER AFFILIATION**	Royal Liverpool and Broadgreen University Hospitals NHS Trust
**REVIEWER CONFLICT OF INTEREST**	None to declare - my centre is a recruiting centre for the study
**DATE REVIEW RETURNED**	23-May-2024

**GENERAL COMMENTS**	This is an important and long overdue study. The protocol and study design has been considered in detail and the long term follow-up is an important element of the study, and having a broad range of possible diagnostic tools I think is the right approach. I also think the focus on aetiologies and risk of developing HCC are important elements given the EASL and AASLD positions on moving to risk based surveillance (without recommending how we might do this).<br><br>I would support acceptance without revision. Only slight quibble would be to change "We" to the Investigators early in the manuscript.

VERSION 1 – AUTHOR RESPONSE

Reviewer 1 (Dr Paul Karanicolas) comments:

1) "*What is the rationale for excluding patients who do not have one of the five specified aetiologies of HCC?"*

- To adequately power the study, we are looking to recruit 3000 patients with liver cirrhosis with aetiologies where the incidence of HCC >1%/year. To ensure we can properly assess and power for the new technologies, models and technique we have thus focused on aetiologies of liver cirrhosis that are the major drivers of HCC.

- When we write the final manuscript for the completed study, we will acknowledge this as a study limitation as our findings will be limited to the patient populations included in our study.

2) *"I'm concerned exclusion criteria #4 "In the view of the clinician, if the patient has a co-morbidity likely to lead to death within the following 12 months" is too subjective and could limit enrolment or generalizability of the ultimate cohort. I understand the rationale, to limit enrolment of participants who will die during the study period and thus not contribute meaningful information to the analysis, but I wonder if the downsides of this criteria outweigh the potential benefit. The same applies to criteria #5: "In the view of the clinician, if the patient is not thought to be suitable for HCC surveillance." At a minimum, participants who are excluded on this basis should be recorded and reported in the ultimate manuscript so that clinicians can interpret the applicability of the findings."*

- We acknowledge that these exclusion criteria are subjective. It is very common for clinical studies, both observational and RCTs, to allow for subjective criteria that aim to ensure only patients will be recruited who are likely to survive the duration of the study. This is intentionally subjective, as objective criteria that define survival for all scenarios do not exist, so by necessity this requires "clinical judgement" in its broadest sense. Furthermore, it is common current practice for clinician judgements like these to determine which patients are included in HCC surveillance programs and hence this somewhat subjective inclusion criteria are representative of real-world practice in HCC surveillance.

- We acknowledge that it will be good to understand which patients have been excluded from the study and we will review whether recording excluded patients would be feasible going forward. We will either include this in the final manuscript or highlight it as a limitation (if not feasible to record excluded patients).

- We currently include eligible patients who have declined to participate in the study in the screening logs and have added a couple of sentences to highlight this.

- We have made the following change to the manuscript
    - Addition of "Patients who are eligible for the study but decline to participate will be logged into the site-specific study logs. This will allow for some level of review and analysis when looking at the final data set." (see page 7 Recruitment).

3) *"The diagnostic criteria for cirrhosis (table 1) seem like they would include a patient with left-sided portal hypertension (for example, a patient with chronic pancreatitis causing splenic vein occlusion and resulting varices). I know this is not the intent, but the authors might consider revising this to avoid inadvertent inclusion of participants without cirrhosis."*

- The presence of liver cirrhosis, aetiology and the method of diagnosis of cirrhosis are key inclusion criteria. Hepatologists will identify definite cases of liver cirrhosis when screening patients for the study and exclude any patients with left sided portal hypertension without liver cirrhosis.

- In light of this comment, we have reviewed all the patients currently recruited to the trial (~1770 participants). We can reassure the reviewer that we have not mistakenly recruited any patients with left sided portal hypertension without cirrhosis.

- Interestingly a significant minority (n=21) of our patients do indeed have portal vein thrombosis but also have concurrent liver cirrhosis.

4) *"The sample size calculation estimates 75 participants developing HCC over the timeframe of study, then goes on to say that allowing 10 events per variable, up to 10 prediction parameters will be included. Shouldn't this read 7 variables to be included, rather than 10? Ideally the authors would state which variables they plan to include in the model, or some description of how they will select the variables. The analysis section in general is very short and lacks many details, this really should be expanded to provide more information on the analytic plan."*

- Thank you for this comment regarding the number of variables to be included, we agree that this needs revising. We have reviewed this with our trial statistician. We believe that there will be 90-180 HCC cases detected (dependent on incidence rate) allowing for 2 years passive follow up. We have added a figure (Figure 4) which highlights the cumulative number of patients with HCC over time (depending on the incidence rate which is not currently fully known). We have now adjusted the number of prediction parameters which may be included in our model. We have clarified and edited the language in the 'Sample Size', 'Statistics & Analysis' and the 'Objectives & Outcome Measures' sections to give further clarity regarding objectives (especially with regards to the secondary objectives and the analysis of them), the anticipated cohort of HCC cases and the subsequent number of variables to be selected dependant on the number of HCC cases detected.

- We agree that, ideally, the variables that will be included in the model would be listed in the protocol. However, we feel that we are unable to do this currently because many of the parameters that will be evaluated for potential inclusion in the model are dependent on a range of exploratory laboratory assays (for example methylation profiles of cell free DNA). This data has not yet been generated and we do not know what will be suitable for model inclusion. We have added a sentence to the protocol to highlight and explain this.

- We have intentionally kept the analysis section short on the protocol manuscript. We are developing a full detailed statistical analysis plan (typically a plan of this depth will be more than 30 pages and would typically not be included in a protocol of this nature). This will be subject to detailed review by the trial management group including external representation.

- We have made the following changes to the manuscript:

    - Clarification of how the ‘prognostic models’ will be evaluated, clarification of the secondary objectives and improved formatting: “A key secondary objective of Pearl is to develop prognostic models for ‘risk-stratifying’ patients with cirrhosis according to their future risk of HCC. This could enable clinicians to personalise HCC surveillance. The models developed will be evaluated primarily in terms of their discriminative ability (i.e. ability to differentiate individuals who develop HCC from those who do not), measured via the Concordance Index (C-Index). In this context, the C-index will indicate the degree to which individuals who develop HCC have a higher risk score than those who do not.

An additional secondary objective includes quantifying the cumulative incidence of HCC among people with cirrhosis and determine how this varies by underlying cirrhosis aetiology in a UK setting. The cumulative incidence of HCC (stratified by HCC aetiology) will be measured at 1-, 3- and 5-years post-baseline.” (see page 10, Objectives & Outcome Measures).

And:

“A key secondary objective is to develop prognostic models (i.e. risk scores or risk calculators) that estimate a cirrhosis patient’s individualised risk of developing HCC in the future. The investigators will approach this in two main ways. First, developing static risk models that incorporate biomarker data collected at a single time point only (i.e. study enrolment). Second, building dynamic models that leverage the serial data collected for Pearl participants in order to estimate individualised HCC risk. As with our primary outcome, our focus will be on developing models that combine multiple prognostic factors/detection assays.” (see page 11-12 Statistics & Analysis)

    - Addition of: “Many of the parameters to be evaluated as part of the model will be selected dependent on data generated from exploratory laboratory assays.” (see page 12, Sample Size).

    - “It is anticipated that this cohort will yield ~90-180 incident HCC cases over, for example, a five-year time frame, depending on the HCC incidence rate observed (Figure 4). Based on the rule-of-thumb of ten events per variable, this would provide scope to include ~10-20 prediction parameters in our model, for this time horizon.” (see page 12, Sample Size).

    - Addition of Figure 4 (see below) to highlight the number of HCC cases to be observed (dependent on the HCC incident rate and time since enrolment). (see page 12, Sample Size):

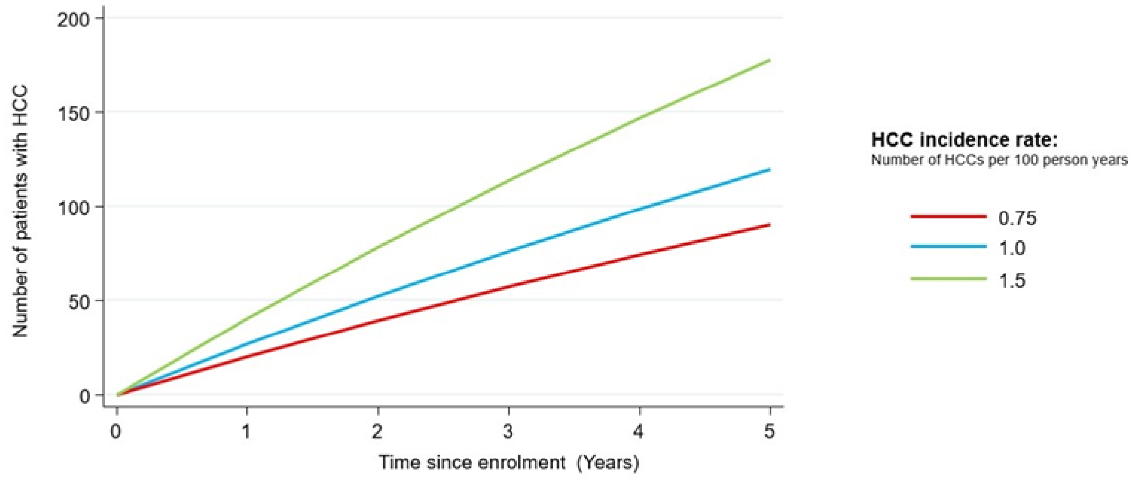

Figure 4: Number of patients expected to develop HCC in the Pearl cohort, according to time since enrolment and HCC incidence rate (N.B. These estimates assume an overall sample size of 3000 patients with 10% attrition. All cause mortality is estimated at 5% per year).

5) *"The study is non-interventional, but this might provide a nice opportunity to identify patients upon diagnosis of HCC who might be candidates for interventional trials. In particular, if patients are found to have more advanced HCC during surveillance (clearly not the intent), there will likely be other interventional trials that they qualify for. The authors might consider working with existing collaborative groups to provide trial opportunities for patients newly diagnosed with HCC as part of the cohort study"*

- We agree with this point completely. This is something that will be looked at going forwards with the study. The chief investigator is currently undergoing discussion with companies developing new immunomodulators for HCC and is seeking collaborations for trial opportunities for patients within the DeLIVER studies.

- We have also recently secured a significant grant from NIHR to perform the AMULET Study on abbreviated MRI vs Ultrasound for the detection of liver cancer (https://oxfordbrc.nihr.ac.uk/new-study-looks-at-new-mri-technique-to-detect-liver-cancer/). Patients in the AMULET study will be recruited from the Pearl cohort. The manuscript has been updated to highlight this change.

- We have made the following changes to the manuscript:

    - Addition of: "A further secondary objective will be utilising the Pearl cohort to identify patients at high risk of developing HCC using the AMAP clinical risk score, for the development of novel MR imaging as part of the separate AMULET study (31)" (see page 10, Objectives and Outcome Measures).

- Addition of another reference: “31. Study assesses new MRI technique to detect liver cancer [Internet]. 2024 [cited 2024 Jul 3]. Available from: https://oxfordbrc.nihr.ac.uk/new-study-looks-at-new-mri-technique-to-detect-liver-cancer/” (see page 16, References).

Reviewer 2 (Dr Tim Cross) comments:

6) *“Change "We” to “the Investigators” early in the manuscript”*

- We have made the following change to the manuscript:
  - Replaced ‘we’ with ‘the investigators’ in the manuscript when appropriate.

Additional changes:

7) Change to Strength and Limitation number 2 and 4 to tighten the language.

- We have made the following change to the manuscript:
  - “The Pearl cohort aims to be representative of liver cirrhosis in the UK.” (see Page 2 Strength and Limitations)

  - “Data and samples will be collected over time via national UK health registries for long-term outcomes. Variable time of sample collection in relation to development of liver cancer.” (see Page 2 Strength and Limitations)

8) On re-review of the manuscript, it was noted a figure had been quoted without sufficient reference (“more than 60,000 people living with cirrhosis”) hence we have provided a reference for this figure.

- We have made the following changes to the manuscript:
  - Addition of reference “In the UK it is estimated that more than 60,000 people have cirrhosis (9)” (see page 4, Introduction).

  - Addition of reference “There are currently more than 60,000 people living with cirrhosis (9)” (see page 12, Sample size).

- Addition of reference: “9. Williams R, Aspinall R, Bellis M, Camps-Walsh G, Cramp M, Dhawan A, et al. Addressing liver disease in the UK: A blueprint for attaining excellence in health care and reducing premature mortality from lifestyle issues of excess consumption of alcohol, obesity, and viral hepatitis. The Lancet. 2014 Nov;384(9958):1953–97. doi:10.1016/s0140-6736(14)61838-9” (see page 15, References).

9) Since original submission of the protocol manuscript, one of the funders is no longer able to contribute and hence one of the DeLIVER Consortium authors is no longer a part of the consortium. Hence his removal from the DeLIVER consortium author list and the removal of the funder (Freenome Holding

- We have made the following change to the manuscript:

  - Removal of Freenome Holdings, Inc as a commercial partner of DeLIVER (see page 14 Funding)

  - Removal of Christopher Welberry from the DeLIVER Consortium author list (see page 20 Appendix 1).

VERSION 2 – REVIEW

**REVIEWER NAME**	Karanicolas, Paul
**REVIEWER AFFILIATION**	Sunnybrook Health Sciences Centre, Surgery
**REVIEWER CONFLICT OF INTEREST**	None
**DATE REVIEW RETURNED**	08-Aug-2024

**GENERAL COMMENTS**	thank you for revising, all comments have been addressed. best of luck with the study.

VERSION 2 – AUTHOR RESPONSE
